# Adherence and Persistence with GLP-1-Based Therapies: International Real-World Evidence and the Role of Nutritional and Lifestyle Support—A Narrative Review

**DOI:** 10.3390/nu18111761

**Published:** 2026-05-30

**Authors:** Artur Dziewierz, Zbigniew Siudak

**Affiliations:** 1 2nd Department of Cardiology, Institute of Cardiology, Jagiellonian University Medical College, 30-688 Krakow, Poland; artur.dziewierz@uj.edu.pl; 2 Clinical Department of Cardiology and Cardiovascular Interventions, University Hospital, 30-688 Krakow, Poland; 3 Collegium Medicum, Jan Kochanowski University, 25-516 Kielce, Poland

**Keywords:** GLP-1 receptor agonists, treatment persistence, medication adherence, medical nutrition therapy, sarcopenic obesity, gastrointestinal tolerability

## Abstract

**Background/Objectives:** Glucagon-like peptide-1 receptor agonists (GLP-1 RAs) have transformed type 2 diabetes mellitus (T2DM) and obesity care, with clinical trials demonstrating weight loss exceeding 15%. However, real-world effectiveness lags trial efficacy, largely owing to high discontinuation rates. We characterize the global persistence gap and propose a framework integrating Medical Nutrition Therapy (MNT) to improve adherence. **Methods:** We conducted a narrative review of real-world evidence from North America, Europe, Asia, and Latin America, synthesized with physiological, nutritional, and behavioral data to distinguish established contributors to discontinuation from strategies that remain partly extrapolated from related populations. **Results:** Global persistence varies widely: from approximately 75–80% at 12 months in reimbursed T2DM cohorts (Sweden, Denmark) to below 10% in obesity-focused or high out-of-pocket-cost settings (Poland, Colombia), with intermediate rates in the United States and United Kingdom; in several cohorts, persistence falls below 15% by 24 months. The primary drivers are gastrointestinal intolerance and economic barriers. Meal size, dietary composition, and gastric-emptying effects influence gastrointestinal tolerability; inadequate protein intake during rapid weight loss raises concern for lean mass loss. **Conclusions:** Pharmacotherapy alone is unlikely to sustain long-term obesity management. Narrowing the persistence gap will require an integrated care model in which structured nutritional support—targeting protein intake, micronutrient density, and gastric-sparing feeding—is systematically offered rather than treated as an optional adjunct, while recognizing that most supporting evidence is extrapolated from primary trials in obesity and cardiometabolic disease rather than derived from GLP-1–specific randomized trials.

## 1. Introduction

The therapeutic landscape for metabolic diseases—encompassing type 2 diabetes mellitus (T2DM), obesity, and their cardiovascular sequelae—has been fundamentally transformed by the widespread adoption of glucagon-like peptide-1 receptor agonists (GLP-1 RAs) [1,2]. From the initial introduction of short-acting agents to the current dominance of long-acting analogues, including semaglutide and the dual glucose-dependent insulinotropic polypeptide (GIP)/GLP-1 RA tirzepatide, these pharmacotherapies have expanded the non-surgical treatment options available for obesity and cardiometabolic disease [3,4,5]. This pharmacological evolution reached a significant policy milestone in December 2025, when the World Health Organization issued its first global guideline conditionally recommending GLP-1 therapies for weight management, formally reinforcing the recognition of obesity as a chronic, relapsing disease requiring sustained, structured care [6].

Randomized clinical trials (RCTs) have consistently demonstrated efficacy profiles previously unattainable with non-surgical interventions [4,7,8]. Foundational studies such as STEP [9,10] and SURPASS [11,12] established high benchmarks for weight reduction, while recent data from the SURMOUNT-5 [13] head-to-head trial demonstrated that dual agonism with tirzepatide can achieve mean body weight reductions exceeding 20%. Furthermore, landmark trials such as SELECT have confirmed significant reductions in major adverse cardiovascular events (MACE), establishing these agents as systemic disease-modifying therapies beyond glycemic and weight control [7,14,15].

However, a critical dichotomy has emerged between the highly supervised environment of RCTs and the realities of routine clinical practice [4,5,16,17,18]. While RCTs report retention rates often exceeding 85% over 68 weeks [9], real-world evidence from diverse healthcare systems—ranging from the insurance-based models of the United States to the single-payer systems of Scandinavia and the United Kingdom—consistently indicates that a substantial proportion of patients discontinue therapy within the first year [16,18,19,20]. This attrition fundamentally undermines the chronic disease management model required for obesity and diabetes care, leading to weight regain, metabolic rebound, and the forfeiture of long-term cardiovascular protection [6,17].

The etiology of this discontinuation is multifactorial, involving socioeconomic barriers, prohibitive out-of-pocket costs, supply chain fragility, and psychological factors, including the resurgence of appetitive drive (commonly termed “food noise”) upon missed doses [21,22]. Yet the dominant physiological driver of discontinuation and non-persistence remains gastrointestinal (GI) intolerance, an intrinsic consequence of GLP-1 receptor activation in the gut and brainstem [23,24]. Rapid pharmacologically induced weight loss is also accompanied by substantial reductions in fat-free mass, raising concerns about sarcopenic obesity, reductions in resting energy expenditure, and post-discontinuation weight regain; whether lean-mass loss itself directly precipitates early treatment discontinuation, however, has not been established [25,26].

The central thesis of this review is that real-world persistence with GLP-1 RA therapy is limited by a confluence of physiological intolerance, economic and structural barriers, and behavioral support gaps, and that structured nutritional and lifestyle support represents a physiologically plausible and clinically necessary framework to narrow the resulting persistence gap, while acknowledging that direct, GLP-1 RA-specific randomized evidence demonstrating persistence improvement remains limited. Building on this thesis, the review provides a comprehensive analysis of the global state of GLP-1 RA adherence, synthesizing real-world data to characterize the magnitude of the persistence gap, and argues that narrowing this gap will require the systematic integration of Medical Nutrition Therapy (MNT) and structured behavioral support into routine GLP-1 RA care, rather than their continued treatment as optional adjuncts to pharmacotherapy [27]. We further argue that the strength of evidence for individual components of this integrated framework varies, and we make explicit throughout the manuscript where recommendations are supported by primary trial data and where they rest on extrapolation from related conditions or on expert consensus. We examine the biochemical and physiological basis for nutritional interventions, exploring how dietary composition modulates GI tolerance, how protein intake timing and quantity influence body composition during rapid weight loss, and how integrated lifestyle support determines long-term therapeutic success. By bridging the disciplines of pharmacotherapy and nutritional science, we propose a comprehensive framework for optimizing clinical and patient-centered outcomes in the GLP-1 era.

## 2. Methods

### 2.1. Study Design and Scope

This study was designed as a narrative review synthesizing the current literature on adherence and persistence with GLP-1 RAs and on the role of nutritional and behavioral interventions in supporting long-term treatment retention. The review was restricted to studies conducted in adult populations (≥19 years); pediatric and adolescent data were excluded from the main analysis because the clinical, regulatory, and behavioral context of GLP-1 therapy in children differs substantially from that in adults and warrants separate evaluation.

### 2.2. Search Strategy

A structured literature search was conducted in PubMed as the primary database, supplemented by directed searches in Embase and Google Scholar and by hand-searching of the reference lists of pivotal trials, key reviews, and consensus documents (Supplementary Material S1). The search covered English-language publications from database inception through 31 January 2026 and combined controlled vocabulary (MeSH) and free-text terms across three domains: (i) drug class (“GLP-1 receptor agonist*,” “semaglutide,” “tirzepatide,” “liraglutide,” “dulaglutide,” “exenatide,” “orforglipron,” and brand names); (ii) outcome (“medication adherence,” “treatment persistence,” “discontinuation,” “real-world evidence,” “proportion of days covered,” “treatment retention”); and (iii) intervention context (“medical nutrition therapy,” “dietary intervention,” “sarcopenia,” “lean mass,” “resistance training,” “gastrointestinal adverse events,” “digital therapeutic,” “behavioral intervention,” “artificial intelligence”).

### 2.3. Inclusion and Exclusion Criteria

Eligible publications included randomized controlled trials (RCTs), prospective and retrospective cohort studies, insurance claims and pharmacy benefit database analyses, national registry studies, systematic reviews and meta-analyses, and consensus documents from major scientific societies, provided they reported data relevant to one or more of the following: efficacy or effectiveness of GLP-1 RA therapy in adults; real-world adherence, persistence, or discontinuation; physiological, economic, or psychological barriers to long-term use; and nutritional, behavioral, or digital interventions intended to support persistence or mitigate adverse effects. Exclusion criteria comprised non-English-language publications, studies conducted exclusively in pediatric populations, single case reports unless illustrating a novel mechanistic concept (e.g., the neuroimaging case study cited in Section 5.3), and non-peer-reviewed grey literature, except where explicitly labeled as company-reported real-world evidence.

### 2.4. Study Selection and Synthesis

**Figure 1:**
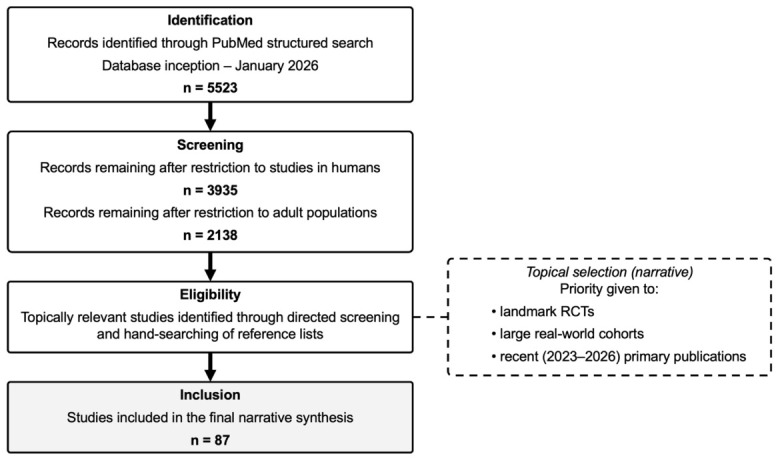
Flow of literature identification and selection for the narrative review.

The primary PubMed search identified 5523 records, of which 3935 remained after restriction to studies in humans and 2138 after further restriction to adult populations (≥19 years). Because this is a narrative rather than a systematic review, comprehensive title-and-abstract screening of all records was not performed; instead, studies were selected for inclusion based on topical relevance to the predefined themes of the review, with priority given to (i) landmark RCTs, (ii) large real-world cohort and claims-database analyses, and (iii) recent (2023–2026) primary publications. Reference lists of pivotal trials, key reviews, and consensus documents were hand-searched to identify additional relevant publications. A total of 87 publications were included in the final narrative synthesis (Figure 1). Two evidence categories were prioritized: (i) RCTs—including landmark programs such as STEP, SURPASS, SURMOUNT, SELECT, and STEP UP—to establish efficacy and safety baselines; and (ii) real-world data from retrospective cohort analyses, insurance claims databases (e.g., Prime Therapeutics, IQVIA), and national registries to quantify effectiveness, persistence, and discontinuation in routine practice. Nutritional and behavioral evidence was drawn from primary trials and cohort studies where available; where direct GLP-1-specific data were lacking, we extrapolated cautiously from primary trials of lifestyle, dietary, and resistance-training interventions in obesity and T2DM, with all such extrapolations labeled explicitly in the text.

### 2.5. Methodological Limitations

Because this is a narrative rather than a systematic review, study selection and synthesis are inherently subject to selection bias, and no formal risk-of-bias assessment, GRADE rating, or meta-analytic synthesis was performed. Persistence definitions, allowable gap thresholds (60 vs. 90 days), follow-up durations, and data sources differ substantially across the cited real-world studies, which preclude direct quantitative cross-cohort comparison. The evidence base is also predominantly derived from high-income settings, limiting generalizability to low- and middle-income countries.

## 3. Physiology of GLP-1 and Nutritional Interaction

Understanding the challenges of adherence and the rationale for nutritional support requires an appreciation of the physiological interplay between exogenous GLP-1 RAs and the endogenous nutrient-sensing machinery of the human body. GLP-1 does not operate in isolation; it both modulates and is modulated by the nutritional environment of the gut [28].

### 3.1. Mechanisms of Action: The Gut–Brain Axis

Endogenous GLP-1 is an incretin hormone secreted by enteroendocrine L-cells in the distal ileum and colon in response to nutrient ingestion [28]. Its half-life is remarkably short (1–2 min) owing to rapid degradation by dipeptidyl peptidase-4. Therapeutic GLP-1 RAs are resistant to this enzymatic degradation, providing supraphysiological levels of receptor activation over extended periods [29,30].

The mechanisms driving weight loss and glycemic control are pleiotropic [29,30]. GLP-1 receptors are densely expressed in the hypothalamus (arcuate nucleus) and the hindbrain (area postrema and nucleus of the solitary tract), where their activation enhances satiety signaling and attenuates the hedonic drive for food [31]. Recent neuroimaging and behavioral studies indicate that GLP-1 RAs also suppress activity within the mesolimbic reward circuitry and modulate the default mode network, effectively reducing maladaptive food-related prospection, commonly described as “food noise” [32]. In addition, GLP-1 inhibits gastric emptying and reduces bowel motility through a vagally mediated gastric braking mechanism [29,30]. While this physically limits food intake by prolonging the sensation of fullness, it is also the principal driver of upper GI adverse events. Furthermore, GLP-1 enhances glucose-dependent insulin secretion from pancreatic beta cells and suppresses glucagon secretion from alpha cells, thereby reducing hepatic glucose output [29,30].

### 3.2. Nutritional Feedback Loops and Adverse Events

The mechanistic discussion in this section synthesizes established physiology of GLP-1 signaling and gastric emptying with the dietary modulation literature in functional dyspepsia and gastroparesis. The specific proposition that distinct dietary patterns (particularly high-volume, high-fat meals) directly exacerbate GLP-1-induced GI symptoms in a clinically meaningful way is grounded in this physiology but has not been formally tested in randomized dietary-modification trials within GLP-1 RA cohorts. Recommendations derived from this mechanistic synthesis should therefore be understood as physiologically plausible expert consensus rather than as RCT-validated interventions.

The interaction between dietary composition and GLP-1 pharmacology is central to tolerability [27,33]. GI side effects—including nausea, vomiting, bloating, and diarrhea—are profoundly dose-dependent and frequently exacerbated by specific macronutrient profiles [27,33]. As demonstrated in the 2025 STEP UP trial, aggressive dose escalation to ultra-high formulations (e.g., semaglutide 7.2 mg) exacts a considerable physiological toll, with GI adverse events affecting over 70% of participants [34].

Dietary lipids are potent stimulators of endogenous gut hormones, including cholecystokinin and peptide YY, which act synergistically with GLP-1 to inhibit gastric emptying [35]. When a patient receiving a high-dose GLP-1 RA consumes a high-fat meal, the combined effect of pharmacological and endogenous lipid-mediated signaling can produce profound gastric stasis [27,33]. This results in prolonged retention of gastric contents, leading to fermentation, distension, and stimulation of mechanoreceptors that trigger the emetic reflex.

The gastric braking effect also reduces the functional capacity of the stomach [36,37]. Patients accustomed to consuming large volumes of low-energy-density foods are likely to exacerbate GLP-1-induced gastric stasis and postprandial discomfort, supporting a shift toward nutrient-dense, lower-volume feeding patterns during the titration phase [27,33,37].

Nutritional counseling must therefore decouple hydration from thirst cues, prescribing fluid intake as a structured daily requirement. Population-level Adequate Intake values established by the U.S. Institute of Medicine (approximately 2.7 L/day for adult women and 3.7 L/day for adult men from all dietary sources) provide a reasonable upper anchor for daily targets [38], with individualized adjustment based on body weight, activity level, climate, and the presence of GI losses [27,33]. Importantly, these targets apply to the general GLP-1 RA population and should not be generalized to patients requiring fluid restriction—including those with congestive heart failure, advanced chronic kidney disease, or decompensated cirrhosis with ascites—in whom individualized fluid targets should be set by the treating cardiologist, nephrologist, or hepatologist rather than by generalized nutritional advice.

## 4. Global Epidemiology of Adherence and Persistence

The gap between clinical efficacy and real-world effectiveness is largely defined by patient adherence and persistence. Adherence refers to conformity with dosing recommendations, often measured by the proportion of days covered (PDC), while persistence refers to the continuity of therapy over time [39]. Global data reveal distinct patterns of utilization and discontinuation across all regions, shaped by healthcare system structure, reimbursement policies, and supply chain dynamics (Table 1). However, the persistence and adherence estimates summarized in Section 4.1, Section 4.2 and Section 4.3 and in Table 1 should be interpreted with appropriate caution. The cited studies draw on heterogeneous data sources that differ substantially in study design, data granularity, and underlying patient populations. Operational definitions of persistence and discontinuation likewise differ across these sources, with allowable gap thresholds ranging from 60 to 90 days, with some studies measuring persistence as a binary outcome at a fixed time horizon and others as a continuous PDC, and with some applying additional eligibility filters such as minimum days of medication supply (Table 1). Real-world datasets are, additionally, subject to potential biases related to insurance coverage, healthcare system structure, and reimbursement policy, which vary markedly across the regions covered in this review. These methodological differences preclude direct quantitative comparison of persistence percentages across cohorts and preclude formal meta-analytic synthesis within the present narrative review. We therefore present these estimates as complementary perspectives on a globally consistent phenomenon, that real-world persistence with GLP-1 RA therapy is substantially below trial-based efficacy, rather than as numerically commensurate values from a unified data source.

### 4.1. North America

In the United States, the uptake of GLP-1 RAs has increased rapidly, yet persistence rates remain low. The market is characterized by high out-of-pocket costs, complex insurance utilization management, and recurrent supply shortages [27,40,41]. Recent longitudinal company-published, non-peer-reviewed data from Prime Therapeutics, analyzing commercially insured individuals initiating GLP-1 therapy for obesity without diabetes, illustrate the depth of the persistence problem. At three years, only 8.1% of patients—fewer than 1 in 12—remained persistent on therapy. The financial implications of this cyclical pattern of initiation and discontinuation are considerable; by early 2025, GLP-1 costs accounted for 15% of the entire commercial pharmacy benefit expenditure, reaching $29.15 per member per month.

Further compounding this issue is pre-initiation attrition. A large-scale 2025 IQVIA analysis revealed that 15% of all approved GLP-1 prescriptions are abandoned at the pharmacy counter without a single fill, while an additional 36% of patients discontinue therapy immediately following their first fill. This rapid abandonment reflects the dual barriers of acute GI intolerance and cost-related deterrence, underscoring the need for robust pre-initiation counseling [42].

Another U.S. cohort study found that 46.5% of patients with T2DM and 64.8% of patients with obesity discontinued treatment within one year [19].

### 4.2. Europe

European data, derived largely from national registries, provide insight into adherence within systems with stricter prescribing indications. Within Sweden, where GLP-1 RA therapy for T2DM is covered within the public reimbursement framework, Lim et al., analyzing 73,895 new users initiated between 2017 and 2021 using a 90-day grace-period definition of discontinuation identical to that used in the Polish data described below, reported a cumulative discontinuation incidence of 23.6% at one year and 38.5% at three years—corresponding to one-year persistence of approximately 76.4% and three-year persistence of approximately 61.5% [43,44]. Notably, among patients who discontinued, 41.1% reinitiated therapy within one year and 57.4% within three years, suggesting that discontinuation in the Swedish context is frequently transient rather than terminal. Recent UK data [45] indicate intermediate persistence in a T2DM cohort (approximately 54.8% at 12 months and 35.3% at 24 months), with a median time to discontinuation of 426 days. Against this Scandinavian and UK background—both characterized by national reimbursement of GLP-1 RA therapy for T2DM—recent data from Poland illustrate the pronounced incremental impact of unsubsidized medication costs.

An analysis of the Polish LUX MED database (n = 34,024 initiating adults; January 2018–May 2024 index period with follow-up through May 2025) characterized persistence through prescription refill patterns operationalized with a >90-day allowable gap [46]. Only 6.6% of patients achieved long-term use (defined as ≥12 consecutive prescriptions, approximately corresponding to 12 months of continuous therapy); 35.1% received only a single prescription; and 58.3% received between 2 and 11 prescriptions before discontinuation. Long-term users (Group 3) were older, more likely to be male, and substantially more likely to have T2DM (55.5% vs. 29.9% in Group 1), suggesting that established cardiometabolic indication and possibly favourable reimbursement (notably the Polish ‘75+’ programme) help to sustain therapy in the small minority who persist. A complementary analysis of the same LUX MED dataset by Dziewierz et al. addressed the upstream question of treatment uptake: among adults without diabetes who were eligible for GLP-1 RA therapy for weight management, only 4.7% actually initiated treatment between 2018 and 2023 [47]. The two analyses therefore draw on the same underlying data source but define and analyze distinct cohorts to address complementary stages of the patient journey—Siudak et al. [46] quantified persistence among adults who had already started therapy, whereas Dziewierz et al. [47] quantified initiation among the broader population eligible to start it. Together, the two analyses indicate that the Polish patient journey is constrained at both ends: most eligible patients never begin therapy, and most of those who do begin discontinue within 12 months.

Comparable nationwide register data from Denmark provide the largest and most methodologically detailed European reference point. Lassen et al., analyzing 44,343 first-time GLP-1 RA users with T2DM initiated between 2007 and 2020 in the Danish nationwide registries using a >90-day gap definition identical to the Swedish and Polish definitions, reported a 12-month discontinuation risk of 21.2% (corresponding to 12-month persistence of 78.8%) and a 6-month discontinuation risk of 14.2% [48]. Notably, while overall persistence was high, only 50.4% of patients met the adherence threshold of PDC ≥0.80 at 6 months and 48.6% at 12 months—indicating that a substantial proportion of patients formally remain on therapy but are intermittently adherent. Lassen et al. also documented a marked socioeconomic gradient: the 12-month discontinuation risk was 24.5% in the lowest household-income tertile compared with 18.0% in the highest, even within a publicly reimbursed system with a capped annual co-payment of approximately USD 630—suggesting that residual cost-sharing alone produces measurable persistence differentials. Increased discontinuation was also observed at the youngest (<40 years; 29.6%) and oldest (>75 years; 27.7%) age extremes and in patients with higher comorbidity burden, the very groups most likely to derive cardio-renal benefit from sustained therapy.

**Table 1:** Global Variations in GLP-1 RA Persistence and Adherence (Selected Studies).

Region/Database	Population	Early Attrition	Long-Term Persistence *	Primary Identified Barriers
USA (Prime Therapeutics/IQVIA)	Obesity without diabetes	15% prescription abandonment at pharmacy; 36% discontinuation after first fill	8.1% at 3 years	High per-member per-month costs; supply chain instability; GI adverse events
United Kingdom (CPRD) [45]	T2DM	Not reported as a discrete 1-month value; median time to discontinuation 426 days	~54.8% at 12 months; ~35.3% at 24 months	GI intolerance; dose-frequency effects (paradoxical higher discontinuation with weekly dosing); modest real-world weight loss
Sweden (nationwide registers) [44]	T2DM	Not reported as a discrete one-month value	~76.4% at 1 year; ~61.5% at 3 years	GI intolerance; out-of-pocket costs (recent cohorts initiating semaglutide for weight loss)
Denmark (nationwide registers) [48]	T2DM	~14.2% discontinuation at 6 months	persistence ~78.8% at 12 months; adherence (PDC ≥ 80%) 48.6% at 12 months	Lower household income, younger (<40 y) and older (>75 y) age, higher comorbidity burden; residual co-payment despite universal reimbursement
Poland (LUX MED) [46]	Mixed	~35.1% discontinuation after a single prescription	6.6% long-term persistence (~6% estimated national penetration)	Absence of state reimbursement for obesity pharmacotherapy; prohibitive out-of-pocket costs
Colombia (nationwide registers) [49]	Mixed	35.4% discontinuation after one month of therapy	13.8% at 6 months; 0.2% at 12 months	Limited access; prohibitive out-of-pocket costs; insufficient clinical follow-up
Saudi Arabia (tertiary-care cohort) [50]	T2DM	Not reported	40% persistence at 1 year	Injection burden; patient preference for oral agents

* Persistence and discontinuation definitions are not standardized across real-world cohorts, which limits direct quantitative comparison of percentages between rows of this table. Operational definitions reported in the source publications are as follows: USA (Prime Therapeutics)-60-day allowable gap between consecutive prescription fills; Poland (LUX MED)-90-day allowable gap between consecutive prescription refills, applied at 12 months of follow-up; Colombia-each prescription assumed to provide 30 days of therapy duration, with discontinuation defined as a 60-day gap from the last prescription date (equivalent to a 30-day allowable gap after the expected supply had ended); Saudi Arabia— persistence defined over a 365-day follow-up as the absence of any refill gap exceeding 60 days; United Kingdom-discontinuation defined as a ≥90-day gap between consecutive prescriptions, with persistence reported at 12 and 24 months; Sweden (Lim et al., nationwide registers)-discontinuation defined as failure to refill before the estimated end date of the most recent prescription with a 90-day grace period; Denmark—discontinuation defined as a gap > 90 days between estimated end of supply and the next prescription refill; adherence operationalized separately as PDC ≥ 80%. Differences in follow-up horizon, allowable gap thresholds, and data source (claims databases vs. registries vs. surveys) should be considered when interpreting cross-cohort comparisons. Importantly, the Prime Therapeutics figures cited in the USA row are company-published real-world analyses that have not been independently peer-reviewed and should be interpreted with corresponding caution; they are presented here alongside, but not as equivalent to, the peer-reviewed registry and claims-database studies that constitute the remaining rows.

### 4.3. Asia and the Middle East

Data from Asia and the Middle East reveal unique adherence patterns influenced by metabolic phenotypes and healthcare access. Evidence from a Saudi tertiary-care cohort indicates that, although GLP-1 RAs are widely prescribed, 12-month persistence with injectable semaglutide is lower than with the oral sodium–glucose cotransporter 2 inhibitor empagliflozin (approximately 40% vs. a higher rate with sodium–glucose cotransporter 2 inhibitors), suggesting that injection burden and cost may drive patients toward oral alternatives [50]. It has been hypothesized that East Asian populations may experience a higher incidence of GI adverse events with fixed-dose GLP-1 RA therapy because of lower average body surface area relative to non-East Asian populations. This proposition is biologically plausible but, in the absence of head-to-head comparative pharmacokinetic and tolerability data, should be regarded as a working hypothesis rather than as an established mechanism. Independent of this question, oral formulations evaluated in East Asian cohorts (e.g., oral semaglutide in the OASIS 2 trial) represent a clinically relevant option for patients who prefer non-injectable therapy [51,52].

### 4.4. Latin America

Data from Latin America reveal profound disparities in access and persistence driven by socioeconomic barriers. A 2025 retrospective study of 9356 new semaglutide users in Colombia documented a rapid decline in the persistence curve. Despite over 90% of patients initiating therapy at the recommended starting doses, 35.4% abandoned treatment after a single month, with a mean duration of use of only 93.7 days. By six months, persistence had fallen to 13.8%, and at 12 months, only 0.2% of the cohort remained on therapy—a figure effectively indistinguishable from zero [49]. This steep decline in persistence demonstrates that, without subsidized healthcare infrastructure and integrated clinical follow-up, the therapeutic potential of GLP-1 RAs is largely negated by economic realities.

## 5. Barriers to Adherence and Persistence with GLP-1 Therapy

The factors driving the persistence gap are complex and interrelated. While clinical trials preferentially enroll highly motivated participants, real-world populations face a combination of physiological, economic, and psychological barriers that frequently undermine long-term therapy [20].

### 5.1. Physiological Barriers and Dose-Escalation Toxicity

GI adverse events remain the most frequently cited medical reason for discontinuation across both clinical trial and real-world settings [24,53]. In the largest cross-sectional survey of GLP-1 RA discontinuation in routine practice to date, both physicians and patients converged on GI symptoms as a leading cause of treatment cessation, although the relative emphasis differed [54]. Surveyed physicians (n = 443) most frequently cited inadequate glycemic control (45.6%), nausea or vomiting (43.8%), and other GI side effects (36.8%) as reasons for stopping therapy in their patients; surveyed patients in turn most frequently cited nausea (64.4%) and vomiting (45.4%) as their principal reasons for abandoning treatment. The pattern indicates that GI intolerance is a dominant driver of discontinuation from both clinical and patient perspectives, with physicians additionally weighing suboptimal therapeutic response and patients additionally weighing symptom severity.

Importantly, discontinuation rates peak during the dose-escalation phase. As higher-dose formulations are developed to maximize weight-loss outcomes, the physiological burden intensifies. This relationship was clearly demonstrated in the 2025 STEP UP trial, which evaluated an investigational 7.2 mg dose of semaglutide [34]. While the ultra-high dose achieved substantial weight loss, GI adverse events occurred in approximately 71% of participants, compared with 61% receiving the standard 2.4 mg dose and 43% receiving placebo. Serious adverse events were reported in 6.8% of the high-dose group [34]. This dose-dependent increase in toxicity suggests that aggressive titration without concurrent MNT to optimize gastric volume management and meal composition substantially increases the risk of early patient discontinuation [27].

### 5.2. Economic and Structural Barriers

The acquisition cost of GLP-1 RA therapy, and the distribution of that cost between patient out-of-pocket expense and payer coverage, are among the strongest determinants of treatment persistence globally. Manufacturer list prices in the United States substantially exceed those in other high-income countries: for semaglutide indicated for obesity (Wegovy 2.4 mg), the monthly U.S. list price of approximately USD 1349 is approximately 4-fold higher than in Germany (USD 328) and the Netherlands (USD 296); for tirzepatide (Mounjaro/Zepbound), the U.S. list price of approximately USD 1023 compares with USD 444 in the Netherlands and USD 319 in Japan; and for semaglutide indicated for T2DM (Ozempic), the U.S. list price of approximately USD 936 is more than 5-fold higher than the Japanese list price of USD 169 and approximately 10-fold higher than corresponding prices in Sweden, the United Kingdom, Australia, and France. A peer-reviewed analysis of estimated minimum production costs suggested that semaglutide could be supplied at approximately USD 40 per 30-day course, indicating that current national prices primarily reflect manufacturer pricing strategy and patent protection rather than cost-of-goods constraints [55]. A recent microsimulation analysis demonstrated that, at current U.S. net prices, tirzepatide and semaglutide for obesity have lifetime incremental cost-effectiveness ratios of approximately USD 197,000 and USD 468,000 per quality-adjusted life-year, respectively—values that would require price discounts of 30.5% (tirzepatide) and 81.9% (semaglutide) to reach the conventional USD 100,000/QALY threshold [56].

In the United States, out-of-pocket costs and utilization management function as direct barriers to persistence. A dose–response relationship exists between cost-sharing and adherence: patients in the highest out-of-pocket cost quartiles have significantly greater odds of nonadherence [57]. A factor unique to the U.S. market is forced discontinuation resulting from mid-year formulary changes or alterations in employer-sponsored coverage, which can interrupt therapy regardless of patient or physician preference [20,41,42]. Even when therapy is initiated, cyclical patterns of initiation and discontinuation impose substantial costs on both patients and payers—illustrated by the rise in GLP-1 expenditure to approximately 15% of total commercial pharmacy benefit expenditure by early 2025, as reported by Prime Therapeutics in U.S. claims data analyzed in Section 4.1.

European reimbursement landscapes are highly heterogeneous, producing markedly different patterns of access and persistence within a single continent. The United Kingdom restricts National Health Service coverage of semaglutide for obesity to specialist weight-management services under NICE Technology Appraisal TA875 [58]; Germany has limited reimbursement following statutory assessment; and several Nordic countries provide partial coverage with patient co-payment, whereas Poland excludes obesity pharmacotherapy from public reimbursement entirely. Because the Polish National Health Fund restricts reimbursement to advanced T2DM and excludes obesity management for patients without diabetes, individuals bear the full monthly cost of approximately EUR 200–250, which translates into unsustainable cumulative expenditure for chronic therapy. The LUX MED database analysis confirmed that, in the absence of state subsidization, 93.4% of patients discontinued therapy within 12 months, with 12-month persistence of only 6.6% [46]. The continuity of this cost-persistence association across very different reimbursement environments is itself instructive. Even within the Danish universal healthcare system, where reimbursable medication is covered at 100% beyond a fixed annual co-payment of approximately USD 630, Lassen et al. demonstrated a 36% higher relative risk of 12-month discontinuation in the lowest household-income tertile compared with the highest [48]. Together with the Sweden–Poland contrast described above and the international list-price disparities documented earlier in this section, these data indicate that GLP-1 RA persistence is driven not by reimbursement status as a binary variable but by the cumulative financial burden borne by the individual patient—a gradient that operates within reimbursed systems and intensifies dramatically in unsubsidized ones.

Cost-driven attrition is not confined to high-income settings. In the Colombian semaglutide cohort, where mean out-of-pocket expenditure exceeded USD 300 per month and most patients lacked supplementary private insurance coverage, persistence fell from 35.4% at one month to 13.8% at six months and 0.2% at twelve months—a trajectory consistent with progressive financial exhaustion rather than therapeutic failure per se [49]. Taken together, data from North America, Europe, and Latin America indicate that the financial environment in which GLP-1 RA therapy is delivered is at least as important a determinant of long-term outcome as the pharmacology itself, and that improvements in real-world persistence will be limited absent structural reforms to drug pricing, reimbursement, and access.

### 5.3. Psychological Barriers and the Transience of Appetitive Drive Suppression

A critical psychological driver of both early treatment success and subsequent relapse is the modulation of appetitive drive, commonly described in patient-reported accounts and the emerging functional neuroimaging literature as “food noise”, a colloquial construct proposed to describe maladaptive prospection and cue-driven mental simulation of short-term food rewards, which preliminary studies have associated with hyperactivity in the default mode network [21]. Activation of central GLP-1 receptors attenuates these dopamine-mediated reward pathways, reducing the incentive salience of food [59].

However, late-2025 functional neuroimaging data have revealed the limitations of this pharmacological effect. A pivotal case study published by investigators at Penn Medicine employed an advanced brain–computer interface to continuously monitor a patient with obesity and binge eating disorder undergoing tirzepatide therapy [32]. Continuous neural recording revealed that, while the dual agonist effectively suppressed signaling within the mesolimbic reward circuitry, this suppression was transient and incomplete.

While these findings are hypothesis-generating rather than definitive, they suggest that GLP-1 therapies may not permanently remodel the deep neural architecture governing impulse control, meaning the pharmacological dampening of the reward pathway could be largely reversible [60]. Consequently, the abrupt resurgence of intrusive food-related cognitions upon a missed dose or discontinuation can be profoundly distressing, frequently precipitating rapid weight regain and a sense of treatment failure [61,62]. These findings support the integration of cognitive behavioral therapy and structured lifestyle interventions as essential components of care, designed to facilitate behavioral retraining while the pharmacological window of reduced appetitive drive remains open.

### 5.4. Clinical Implications for Adherence Management

The barriers described in Section 5.1, Section 5.2 and Section 5.3 translate into the following practical considerations for clinical practice:

- At initiation, anticipated GI symptom burden should be discussed proactively with patients, with explicit guidance that approximately two-thirds of patients receiving standard-dose GLP-1 RAs will experience nausea, and that symptom severity is dose-dependent and typically peaks during titration.
- Dose escalation should be paced individually rather than mechanically; tolerability—rather than a fixed protocol—should determine the speed of titration, with explicit permission for patients and clinicians to extend titration intervals or to maintain therapy at the lowest effective dose.
- The financial trajectory of therapy should be discussed at initiation, including out-of-pocket cost, the consequences of insurance coverage changes, and the realistic expectation of chronic rather than time-limited therapy.
- Psychological and social barriers, including weight stigma, stigma associated with injectable therapy, and changes in social eating patterns, should be addressed proactively as part of routine follow-up rather than waiting for patient disclosure.

## 6. Nutritional Support Strategies

The synthesis of global epidemiological data and physiological barriers leads to a clear conclusion: pharmacotherapy alone is unlikely to be sufficient for long-term treatment retention in most patients [27,33]. Nutritional support represents the primary modifiable intervention for mitigating the physiological barriers to adherence and promoting sustainable changes in body composition.

### 6.1. The Academy of Nutrition and Dietetics Position Statement and Pharmacoeconomic Implications

In a position paper published in late 2025, the Academy of Nutrition and Dietetics stated that all individuals receiving obesity pharmacotherapy should have concurrent access to evidence-based MNT delivered by a registered dietitian nutritionist (RDN) [27]. The integration of MNT extends beyond symptom management and may confer pharmacoeconomic value. Direct evidence quantifying the cost benefit of MNT in patients receiving GLP-1 RA therapy is currently limited; however, in the most closely related population—adults with dyslipidemia and cardiometabolic risk factors—a systematic review and meta-analysis of multiple individual MNT sessions delivered by RDN demonstrated clinically meaningful improvements in lipids, glycemia, body mass index, and blood pressure, together with reduced need for lipid-lowering medications and net cost savings primarily driven by reduced medication use [63]. A more recent systematic review and meta-analysis confirmed and extended these findings [64]. By analogy, MNT integration into GLP-1 RA care may be expected to yield similar reductions in concurrent cardiometabolic medication use, although confirmatory studies in this specific population are needed.

Despite these demonstrated benefits, a significant structural barrier exists in the United States: the Centers for Medicare & Medicaid Services currently reimburses MNT only for patients with pre-existing T2DM or chronic kidney disease, explicitly excluding obesity management in the absence of these comorbidities. This coverage gap creates a paradox in which patients receive highly potent pharmacotherapies without financial access to the nutritional expertise required to use them safely and effectively [65].

### 6.2. Managing Gastrointestinal Toxicity Through Dietary Modification

Dietary composition is one of the most readily modifiable contributors to GI side effects (Table 2), and a GLP-1-compatible dietary approach is grounded in gastric-emptying kinetics. Transitioning from three large meals to five or six smaller, nutrient-dense feedings is hypothesized to reduce mechanical distension of the stomach and thereby attenuate vagal stimulation of the emetic center [27,33]. Because dietary lipids act synergistically with GLP-1 to inhibit gastric emptying, reducing fat load per meal is a physiologically plausible and commonly recommended strategy to reduce postprandial nausea and fullness during GLP-1 RA titration, although randomized dietary-modification trials specifically in GLP-1-treated cohorts are lacking [66,67]. During acute nausea episodes, particularly during dose escalation, the transition to liquid or semi-solid foods may facilitate gastric transit.

### 6.3. Preserving Lean Mass: The Protein Imperative

The rapid weight loss induced by high-dose GLP-1 RAs is accompanied by substantial reductions in fat-free mass, which may impair muscle function and predispose to clinical sarcopenia, particularly in older adults and those with baseline sarcopenic obesity. Reduction in fat-free mass during weight loss is not equivalent to sarcopenia, which is a clinical syndrome defined by reduced muscle strength or physical performance in addition to low muscle mass; nevertheless, preserving fat-free mass during pharmacologically induced weight loss remains a clinically important goal. The standard population-level Recommended Dietary Allowance (0.8 g/kg/day) was derived from nitrogen-balance studies in healthy younger adults under weight-maintenance conditions and is insufficient for patients in a substantial catabolic state, in older adults, or in those with chronic disease—populations that overlap substantially with GLP-1 RA users. The PROT-AGE international position paper, synthesizing nitrogen-balance and clinical-outcome studies, recommends a minimum daily intake of 1.0–1.2 g/kg/day for healthy older adults and 1.2–1.5 g/kg/day for those with acute or chronic illness [68]; corresponding ESPEN consensus recommendations align closely [69]. In the specific context of intentional weight loss, mechanistic and clinical evidence summarized by Cava et al. supports protein intakes at the upper end of this range (≥1.2 g/kg/day, and up to approximately 1.6 g/kg/day in those undergoing significant caloric restriction or rapid weight loss), in combination with resistance exercise, to attenuate the disproportionate loss of fat-free mass that otherwise accompanies negative energy balance [70]. Recent GLP-1-specific narrative consensus extends these recommendations to patients receiving incretin-based pharmacotherapy [71]. For individuals with class II or III obesity, this calculation should be based on adjusted or ideal body weight rather than actual body weight, to avoid physiologically excessive protein prescriptions; for patients with chronic kidney disease (particularly eGFR < 30 mL/min/1.73 m^2^) or advanced liver disease, protein targets must be strictly individualized in consultation with renal or hepatology specialists [68,72].

Because GLP-1 RAs substantially suppress appetite, patients frequently struggle to meet these elevated targets. Implementation of the “protein-first” approach—instructing patients to consume the protein component of their meal before carbohydrates or vegetables—is a practical clinical strategy to optimize muscle protein synthesis within the constraints of reduced overall intake. This approach is supported by the broader food-first framework for post-exercise muscle protein synthesis [73] and by evidence that distributing protein intake of approximately 25–30 g across each of three to four daily meals maximizes the muscle protein synthetic response in adults [68,70].

### 6.4. Micronutrient Density and Deficiency Risks

The substantial reduction in total food volume inherently increases the risk of micronutrient insufficiency. Furthermore, patients frequently gravitate toward simple, low-nutrient foods to manage nausea, further displacing nutrient-dense options. Consequently, patients are at heightened risk for deficiencies in iron, vitamin B_12_, calcium, vitamin D, and zinc [74]. Baseline screening for B_12_, vitamin D, and iron should be considered, especially for those with pre-existing deficiencies or a history of bariatric surgery. A nutrient-dense dietary approach, emphasizing maximum micronutrient content per calorie, combined with routine supplementation with a high-quality multivitamin, is recommended as a standard component of care [27,33,66].

### 6.5. Gut Microbiota Modulation and Postbiotics

Emerging evidence suggests that gut microbiota composition plays a bidirectional role in GLP-1 efficacy and tolerability. A 60-day RCT investigated the targeted use of postbiotic and antioxidant supplementation in 25 adults with obesity. The study demonstrated that supplementation with the yeast *Saccharomyces boulardii* combined with superoxide dismutase resulted in significant reductions in total body weight, fat mass, fasting insulin, uric acid, and the HOMA-IR index [75]. Notably, this weight loss was achieved alongside the preservation of fat-free mass compared with placebo. The investigators also observed a significant decrease in endogenous GLP-1 levels alongside an increase in vitamin D concentrations in the intervention group. These preliminary findings suggest that restoration of intestinal barrier integrity and improvement in systemic insulin sensitivity through microbiome modulation may optimize metabolic homeostasis during pharmacologically induced weight loss. Given the small sample size (n = 25), short duration (60 days), and single-center design of the only available randomized trial, these data should be regarded as exploratory and hypothesis-generating rather than definitive, and require confirmation in larger and independent cohorts before any clinical recommendation can be made.

### 6.6. Strength of Evidence and Methodological Limitations

The Nutritional Support Strategies described in Section 6.1, Section 6.2, Section 6.3, Section 6.4 and Section 6.5 are supported by evidence of varying strength, and we make this hierarchy explicit before discussing complementary behavioral and digital interventions in Section 7. The clearest support is for the broad recommendation to combine pharmacological weight loss with structured dietary and behavioral support: pivotal randomized trials of GLP-1 RAs for obesity (STEP, SURMOUNT, SCALE) all incorporated intensive lifestyle interventions modeled on Diabetes Prevention Program-style curricula, and the magnitude of weight loss in the control arms of these trials confirms that structured lifestyle support produces clinically meaningful effects in this population even without GLP-1 RA therapy [9,13,76,77]. The recommendation to integrate MNT and behavioral support into routine care therefore rests on extrapolation from these trials and from the broader MNT literature in dyslipidemia, obesity, and T2DM [63,64,68,70], rather than from randomized comparisons of GLP-1 RA therapy with versus without formal MNT in real-world settings, which are presently lacking.

Specific recommendations vary in their evidentiary support. Recommendations for elevated protein intake (1.2–1.5 g/kg/day in adults with chronic disease, with higher targets up to ~1.6 g/kg/day during active weight loss), combined with resistance training, are supported by primary trial data and aligned international consensus statements addressing protein requirements in older adults, in chronic disease, and during intentional weight loss [68,69,70,78], even though no trial has tested these specific targets in GLP-1 RA users. Recommendations for nutrient-dense, lower-volume feeding patterns and the practical management of GI side effects are supported by GLP-1-specific narrative consensus and by the broader functional dyspepsia and gastroparesis nutrition literature, but should be regarded as expert consensus rather than as RCT-validated. Recommendations regarding micronutrient screening and supplementation are supported by recent large cohort data (n = 461,382) demonstrating an increased incidence of nutrient deficiencies in GLP-1 RA users [79], although the comparative effectiveness of specific screening or supplementation protocols has not been formally evaluated.

Where the evidence base is weakest, we have flagged the discussion accordingly. The microbiota modulation findings discussed in Section 6.5 derive from a single 60-day randomized trial in 25 adults and should be regarded as exploratory and hypothesis-generating until confirmed in larger independent cohorts. The neuroimaging case study discussed in Section 5.3 illustrates a mechanistic concept but is, by design, a single-patient observation. Finally, the proposed dietary mechanism by which volume-based feeding patterns exacerbate GLP-1-induced gastric stasis is grounded in established physiology but has not been formally tested in randomized trials of dietary modification within GLP-1 RA cohorts. In summary, although the conceptual framework proposed here is consistent with current pathophysiological understanding and with extrapolated evidence from related populations, GLP-1 RA-specific randomized trials of integrated MNT and behavioral support remain an important research priority.

### 6.7. Clinical Implications for Nutritional Support

**Figure 2:**
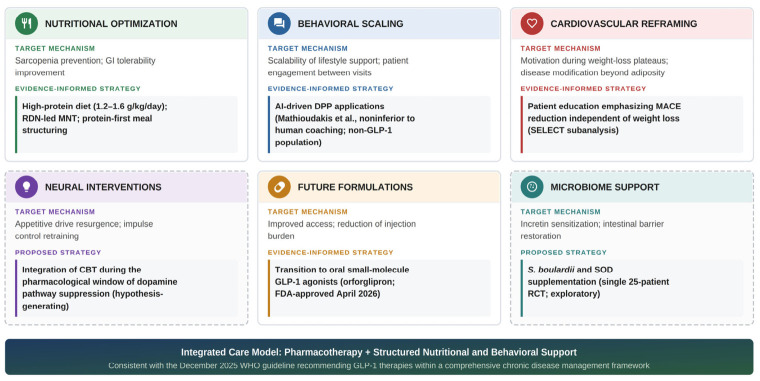
Proposed integrated care framework for closing the GLP-1 RA persistence gap. The framework comprises six strategic domains targeting the principal physiological, behavioral, and systemic drivers of treatment discontinuation, with each domain specifying a target mechanism and an evidence-informed or proposed support strategy. Solid borders denote evidence-informed strategies supported by primary trial evidence or aligned international consensus, even if extrapolated to the GLP-1 RA context. Dashed borders denote proposed strategies that are physiologically plausible and supported by mechanistic reasoning or preliminary data but await confirmation in GLP-1 RA-specific randomized trials; Section 6.6 provides the full strength-of-evidence hierarchy. The framework presents the conceptual scope of integrated care; the temporal sequence in which these components are operationalized across the patient journey is presented separately in Figure 3. AI, artificial intelligence; CBT, cognitive behavioral therapy; DPP, Diabetes Prevention Program; FDA, Food and Drug Administration; GLP-1 RA, glucagon-like peptide-1 receptor agonist; GI, gastrointestinal; MACE, major adverse cardiovascular events; MNT, Medical Nutrition Therapy; RCT, randomized controlled trial; RDN, registered dietitian nutritionist; SOD, superoxide dismutase; WHO, World Health Organization.

**Figure 3:**
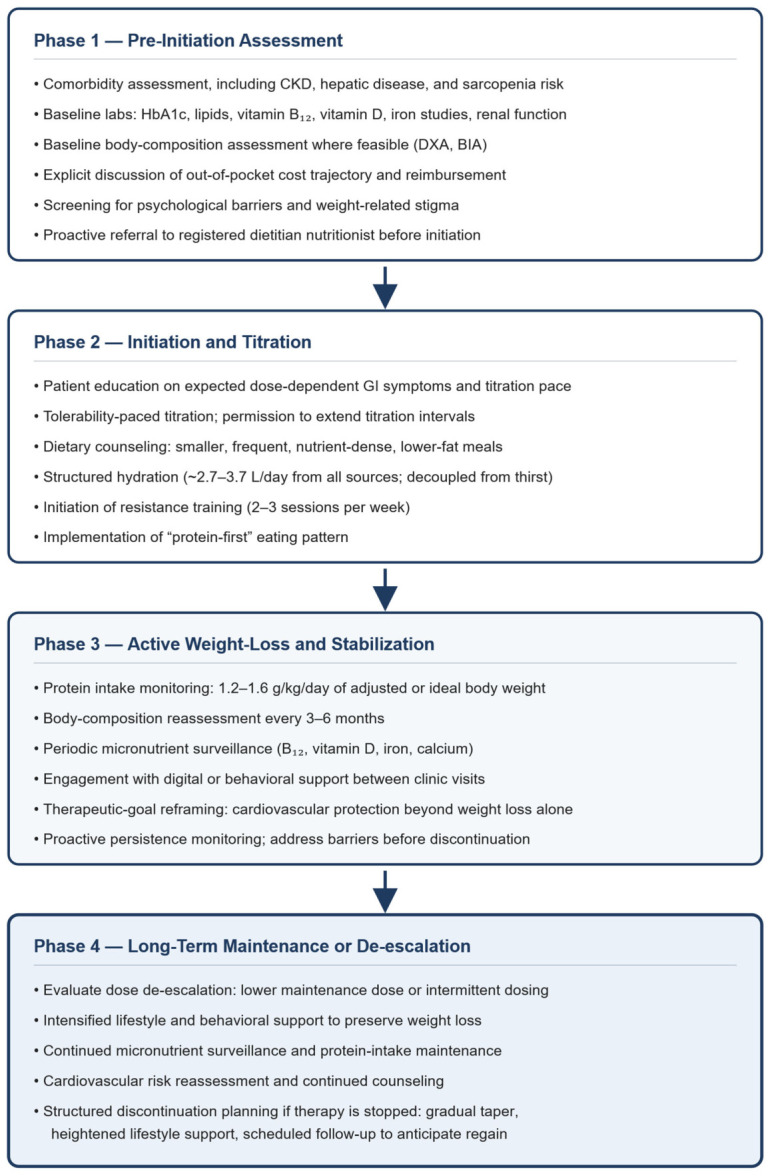
Phase-based clinical pathway for integrated GLP-1 RA care. Each of four sequential phases-from pre-initiation assessment through long-term maintenance or de-escalation-specifies the principal nutritional, behavioral, and clinical actions required at that stage. The pathway operationalizes the components of integrated care (Figure 2) into a temporally sequenced implementation guide for use in routine clinical practice. BIA, bioelectrical impedance analysis; CKD, chronic kidney disease; DXA, dual-energy X-ray absorptiometry; GI, gastrointestinal; HbA1c, glycated hemoglobin.

The nutritional support strategies described in Section 6.1, Section 6.2, Section 6.3, Section 6.4 and Section 6.5 translate into the following practical considerations for clinical practice (Figure 2 and Figure 3):

- All patients initiating GLP-1 RA therapy should be offered referral to RDN where available, with the dietary consultation occurring proactively before or at therapy initiation rather than reactively after the development of GI symptoms.
- Baseline body composition assessment (where feasible) and targets for protein intake (1.2–1.6 g/kg/day of adjusted or ideal body weight, individualized for renal and hepatic comorbidities), distributed across three to four daily meals, should be incorporated into the initial care plan.
- Resistance training of major muscle groups two to three times weekly should be prescribed alongside the pharmacological intervention, particularly during the active weight-loss phase, to mitigate the loss of fat-free mass and the decline in resting energy expenditure that accompany rapid weight loss.
- Baseline screening for iron, vitamin B_12_, vitamin D, and calcium status-and ongoing surveillance during therapy-should be considered in patients with pre-existing deficiencies, restrictive eating patterns, or a history of bariatric surgery.
- Patients should receive practical dietary counseling about volume- and lipid-aware eating, intentional hydration that decouples fluid intake from thirst, and the management of common GI side effects (Table 2).

## 7. Lifestyle and Behavioral Interventions

While nutritional strategies address the physiological side effects of GLP-1 RAs, behavioral interventions target the psychological barriers and habit-formation processes critical for long-term persistence [80,81,82].

### 7.1. Adapting Intensive Lifestyle Intervention Protocols

The most successful weight-loss trials to date—including the STEP program for semaglutide [9] and the SURMOUNT program for tirzepatide [13]—have incorporated intensive lifestyle interventions as a core component of the trial protocol, modeled on the structured behavioral curricula pioneered by the Diabetes Prevention Program (DPP) [76,77]. In these trials, the comparator arm typically received the same lifestyle intervention without active GLP-1 RA therapy, indicating that the GLP-1 RA effect is additive to, rather than independent of, structured behavioral support. In contrast, most patients in routine clinical practice receive a prescription with minimal or no formal behavioral support—a discrepancy that likely contributes to the efficacy–effectiveness gap and to the steep early attrition described in Section 4. Randomized evidence directly comparing GLP-1 RA therapy combined with a formal DPP-style program versus GLP-1 RA therapy alone in real-world settings is currently limited; the recommendation to integrate structured lifestyle and behavioral interventions into routine GLP-1 RA care therefore rests on extrapolation from the cumulative trial evidence rather than on direct head-to-head demonstration.

### 7.2. The Role of Resistance Training

Exercise is among the most important modifiable factors for weight maintenance and body composition preservation. Resistance training directly counteracts the disproportionate loss of fat-free mass and the decline in resting energy expenditure (adaptive thermogenesis) that accompany rapid weight loss [25,70,78]. Furthermore, significant weight loss can decrease bone mineral density; weight-bearing and resistance exercises are therefore essential for the prevention of osteopenia and sarcopenia in this population. Current recommendations emphasize two to three sessions of resistance training per week targeting major muscle groups, alongside standard aerobic activity.

### 7.3. Digital Therapeutics and AI-Driven Lifestyle Support

Given the global shortage of specialized dietitians and behavioral therapists, digital platforms have been proposed as a scalable strategy for delivering structured behavioral support to patients on GLP-1 RA therapy. Company-published real-world data from the Omada Health Enhanced GLP-1 Care Track, analyzing 1124 self-enrolled commercially insured adults without diabetes, reported retention rates of 94% at 12 weeks and 84% at 24 weeks, with mean weight loss of 12.1% among members who persisted through the 24-week program compared with 7.4% among those who discontinued early [83]. These outcomes substantially exceed published real-world persistence estimates, but the underlying cohort is self-selected, digitally engaged, and predominantly commercially insured, which limits direct comparability with the broader real-world populations described in Section 4 and substantially complicates causal inference about the digital-support intervention itself. The data have not, to our knowledge, been independently peer-reviewed.

In a separately designed RCT, Mathioudakis et al. (2025) compared a fully autonomous artificial intelligence (AI)-driven lifestyle-intervention application against traditional human coaching within a digital Diabetes Prevention Program (d-DPP) framework, in adults with prediabetes, overweight, or obesity [84]. Importantly, the trial was conducted in a non-GLP-1-using population, and its direct applicability to GLP-1 RA persistence is by analogy rather than direct demonstration. Within the d-DPP context, the AI-driven intervention was statistically noninferior to human-led coaching for weight loss and glycated hemoglobin reduction, and outperformed human coaching on engagement metrics, with higher rates of program initiation (93.4% vs. 82.7%) and overall completion (63.9% vs. 50.3%) [84]. Together, these emerging data suggest that scaled digital and AI-assisted lifestyle support is a plausible—though not yet definitively proven—strategy for supporting GLP-1 RA persistence in routine care.

## 8. Cardiovascular Prevention: Reframing the Therapeutic Goal

A critical strategy for improving patient persistence is reframing the therapeutic objective from weight loss alone to cardiovascular risk reduction [7,14,15].

### 8.1. Dissociating MACE Reduction from Adiposity: Insights from the SELECT Trial

The SELECT trial demonstrated a 20% reduction in MACE among patients with obesity and established cardiovascular disease [15,85]. A prespecified subanalysis of this cohort, published in The Lancet in late 2025, provided important mechanistic insight [85]. The analysis suggested that the cardiovascular benefit was not fully explained by the magnitude of total weight loss achieved: reductions in waist circumference mediated only approximately one-third of the treatment effect. Although mediation analysis cannot prove complete causal independence from adiposity, these data indicate that an important component of the cardiovascular benefit operates through pathways beyond weight loss alone [85,86].

### 8.2. Pleiotropic Mechanisms and Implications for Adherence

These data indicate that GLP-1 RAs function primarily as direct vascular and systemic disease-modifying agents, operating through anti-inflammatory pathways and endothelial stabilization, rather than solely through adiposity reduction [85,87]. From a clinical counseling perspective, this distinction is of considerable importance. Educating patients that the medication confers cardiovascular and cerebrovascular protection independently of the magnitude of weight loss may provide the necessary motivation to maintain adherence through inevitable weight-loss plateaus.

## 9. Conclusions

The global evidence consistently demonstrates that, while GLP-1 RAs are highly effective agents, their real-world impact is substantially diminished by poor long-term persistence. The attrition of patients—driven by GI intolerance, prohibitive costs, and insufficient behavioral support infrastructure—represents a significant inefficiency in healthcare delivery.

Narrowing this gap will require a shift from a prescription-centered model to an integrated care approach (Figure 2), operationalized as a phase-based clinical pathway (Figure 3). Consistent with the December 2025 World Health Organization guidelines, obesity should be managed as a chronic, relapsing disease in which pharmacotherapy is explicitly paired with evidence-based dietary guidance and structured lifestyle support. MNT should be systematically offered as a standard component of care rather than an optional adjunct, applied proactively to mitigate side effects and reduce the risk of sarcopenia. Protein intake should be prescribed with individualized targets and monitored with the same rigor applied to glycated hemoglobin, and scalable behavioral health interventions, including AI-driven digital therapeutics, should be integrated to maintain therapeutic continuity between clinic visits. Equally important, the therapeutic objective should be reframed from weight reduction alone to comprehensive cardiovascular risk reduction and long-term health gain.

To fully realize the potential of GLP-1 therapies, systemic changes are necessary. Payers and health systems should incentivize and reimburse the support services that promote medication persistence, recognizing that the considerable cost of these agents is largely forfeited if patients discontinue therapy within the first year. Future research into dose de-escalation protocols—examining whether patients can sustain metabolic benefits on lower maintenance doses or intermittent dosing regimens combined with intensive lifestyle interventions—is warranted.

The pharmacological pipeline is also evolving to address adherence barriers directly. Recently approved or emerging daily oral small-molecule GLP-1 agonists, such as orforglipron, may improve access and reduce injection burden [88]. Concurrently, following the termination of one phase 2b trial in patients with obesity and diabetes, other combination trials testing bimagrumab (an activin type II receptor antibody) in non-diabetic obesity remain ongoing, representing a vital avenue for actively preserving lean mass during weight reduction [26].

In summary, this review demonstrates that while the pharmacology of metabolic disease management has advanced considerably, the care delivery systems required to support long-term treatment retention remain underdeveloped. The next decade must prioritize the construction of nutritional and behavioral infrastructure capable of supporting these therapies and closing the persistence gap.

## Figures and Tables

**Table 2:** Nutritional Management of Common GLP-1 Side Effects.

Adverse Event	Physiological Mechanism	Targeted Nutritional/Behavioral Intervention
Nausea	Delayed gastric emptying; central chemoreceptor stimulation	Small, frequent meals; ginger-based beverages; avoidance of high-fat and fried foods; slow eating pace
Vomiting	Gastric overdistension; olfactory triggers	Cessation of eating at early satiety cues; preference for cold-temperature foods; use of liquid-based caloric sources
Sulfurous Eructation/Gastroesophageal Reflux	Gastric stasis with prolonged fermentation and hydrogen sulfide gas production	Selective reduction of sulfur-rich foods (eggs, red meat, cruciferous vegetables) when symptoms are clearly food-triggered, balanced against the need to maintain protein and micronutrient intake; avoidance of carbonated beverages; upright positioning for ≥2 h postprandially
Constipation	Reduced bowel motility; secondary reduction in fluid intake due to suppressed thirst perception	Gradual increase in dietary fiber; structured hydration targets (≥2.0–3.7 L/day); individualized in patients on fluid restriction
Diarrhea	Rapid intestinal transit during early dose titration; fat malabsorption	Low-residue diet (bananas, rice, applesauce, toast); avoidance of sugar alcohols and excessive dietary fat
Fatigue/Sarcopenia	Severe caloric deficit; inadequate protein intake; lean mass catabolism	Protein prioritization at each meal; assessment of vitamin B_12_ and iron status; maintenance of minimum daily energy intake threshold

## Data Availability

No new data were created.

## Supplementary Materials

The following supporting information can be downloaded at https://www.mdpi.com/article/10.3390/nu18111761/s1. Supplementary Material S1—Full PubMed search strategy and record counts at each stage of literature selection.

## Abbreviations

The following abbreviations are used in this manuscript:

AI	Artificial intelligence
BIA	Bioelectrical impedance analysis
CKD	Chronic kidney disease
DPP	Diabetes Prevention Program
DXA	dual-energy X-ray absorptiometry
FDA	Food and Drug Administration
GI	Gastrointestinal
GIP	Glucose-dependent insulinotropic polypeptide
GLP-1	Glucagon-like peptide-1
HbA1c	Glycated hemoglobin
MACE	Major adverse cardiovascular events
MNT	Medical nutrition therapy
PDC	Proportion of days covered
RA	Receptor agonist
RCT	Randomized controlled trial
RDN	Registered dietitian nutritionist
SOD	Superoxide dismutase
T2DM	Type 2 diabetes mellitus
WHO	World Health Organization
