# Supplementary material for: Adherence and Persistence with GLP-1-Based Therapies: International Real-World Evidence and the Role of Nutritional and Lifestyle Support—A Narrative Review
Source: Nutrients. 2026 May 30;18(11):1761. doi: 10.3390/nu18111761 (PMC13259006; doi:10.3390/nu18111761)
Supplement: Supplementary file 1 [file nutrients-18-01761-s001.zip › nutrients-4186202-supplementary.pdf]

Supplementary Material S1

Full PubMed search strategy and record counts at each stage of literature selection

Companion supplementary material to the manuscript "*Adherence and Persistence with GLP-1–Based Therapies: In-ternational Real-World Evidence and the Role of Nutritional and Lifestyle Support—A Narrative Review*" (see Section 2, Methods, and Figure 1).

1. Search execution

- Primary database: PubMed (U.S. National Library of Medicine).
- Supplementary databases: Embase and Google Scholar (used for directed searches and to identify additional pivotal references).
- Search executed: 31 Jan 2026.
- Publication date range covered: from database inception through 31 January 2026.
- Language restriction: English.
- Species restriction: Humans (applied via the PubMed sidebar filter).
- Age restriction: Adult ≥19 years (applied via the PubMed sidebar filter).

Rationale for publication-date cutoff. The cutoff of 31 January 2026 was fixed at the time of the initial manuscript submission and was retained in the revised manuscript to ensure that the cited literature is internally consistent with the search reported. The search was re-executed during preparation of the revised manuscript to verify reproducibility of the record counts; the publication-date filter ensured that no literature published after 31 January 2026 was inadvertently included.

2. Full PubMed search string

The full search string entered into the PubMed search box is reproduced verbatim below. The query combines three domains using Boolean operators: (i) drug class, (ii) outcome (adherence, persistence, discontinuation), and (iii) intervention context (nutritional, behavioral, and digital interventions). Where MeSH terms were available, both controlled-vocabulary and free-text variants were included to maximize sensitivity. Filters for Humans and Adult ≥19 years were applied via the PubMed sidebar after running the query, in order to keep the documented search string concise and reproducible.

```
(
  "Glucagon-Like Peptide-1 Receptor Agonists"[Mesh] OR
  "Glucagon-Like Peptide-1 Receptor"[Mesh] OR
  "Glucagon-Like Peptide 1"[Mesh] OR
  "Semaglutide"[Mesh] OR
  "Liraglutide"[Mesh] OR
  "Exenatide"[Mesh] OR
  "GLP-1 receptor agonist*"[tiab] OR
  "GLP-1 RA"[tiab] OR "GLP-1RA"[tiab] OR
```

```
    "GLP-1 analog*"[tiab] OR "GLP-1 analogue*"[tiab] OR
    "GLP1 receptor agonist*"[tiab] OR
    "incretin mimetic*"[tiab] OR
    semaglutide[tiab] OR tirzepatide[tiab] OR liraglutide[tiab] OR
    dulaglutide[tiab] OR exenatide[tiab] OR lixisenatide[tiab] OR
    orforglipron[tiab] OR retatrutide[tiab] OR
    Wegovy[tiab] OR Ozempic[tiab] OR Rybelsus[tiab] OR
    Mounjaro[tiab] OR Zepbound[tiab] OR
    Saxenda[tiab] OR Victoza[tiab] OR Trulicity[tiab] OR Byetta[tiab]
  )

  AND

  (
    (
      "Medication Adherence"[Mesh] OR
      "Patient Compliance"[Mesh] OR
      "Treatment Adherence and Compliance"[Mesh] OR
      adherence[tiab] OR adheren*[tiab] OR
      compliance[tiab] OR
      persistence[tiab] OR persisten*[tiab] OR
      discontinuation[tiab] OR discontinu*[tiab] OR
      "treatment retention"[tiab] OR
      "real-world"[tiab] OR "real world"[tiab] OR
      "proportion of days covered"[tiab] OR PDC[tiab] OR
      "medication possession ratio"[tiab] OR
      "claims database"[tiab] OR registry[tiab]
    )
    OR
    (
      "Diet Therapy"[Mesh] OR
      "Nutrition Therapy"[Mesh] OR
      "Nutritional Support"[Mesh] OR
      "Sarcopenia"[Mesh] OR
      "Resistance Training"[Mesh] OR
      "Behavior Therapy"[Mesh] OR
      "Cognitive Behavioral Therapy"[Mesh] OR
      "Telemedicine"[Mesh] OR
      "Mobile Applications"[Mesh] OR
      "Artificial Intelligence"[Mesh] OR
      "medical nutrition therapy"[tiab] OR MNT[tiab] OR
      "dietary intervention*"[tiab] OR "nutritional intervention*"[tiab] OR
      sarcopenia[tiab] OR "sarcopenic obesity"[tiab] OR
      "lean mass"[tiab] OR "fat-free mass"[tiab] OR "muscle mass"[tiab] OR
      "resistance training"[tiab] OR "strength training"[tiab] OR
      "gastrointestinal adverse event*"[tiab] OR
      "gastrointestinal side effect*"[tiab] OR
      "gastrointestinal toleran*"[tiab] OR
      "gastric emptying"[tiab] OR
      "behavioral intervention*"[tiab] OR "behavioural intervention*"[tiab] OR
      "lifestyle intervention*"[tiab] OR "lifestyle modification"[tiab] OR
      "digital therapeutic*"[tiab] OR "digital health"[tiab] OR
```

```
    telehealth[tiab] OR telemedicine[tiab] OR
    "artificial intelligence"[tiab] OR "machine learning"[tiab]
  )
)

AND English[lang]
AND ("1900/01/01"[Date - Publication] : "2026/01/31"[Date - Publication])
```

3. Record counts at each stage of selection

Counts at each stage of the literature selection process are summarized in Table S1.1 and visualized in Figure 1 of the main manuscript.

Stage	Filter applied	Records (n)
Identification	PubMed structured search; publication date up to 31 January 2026; English language	5,523
Screening	Records remaining after restriction to studies in humans (PubMed sidebar filter)	3,935
Eligibility	Records remaining after further restriction to adult populations ≥19 years (PubMed sidebar filter)	2,138
**Inclusion**	Topically relevant studies identified through directed screening and hand-searching of reference lists; included in narrative synthesis	**87**

Table S1.1. *Record counts at successive stages of literature selection. The Humans and Adult ≥19 years restrictions were applied as PubMed sidebar filters after the structured search string in Section 2 had been executed.*

4. Inclusion and exclusion criteria

Inclusion criteria. Eligible publications comprised randomized controlled trials, prospective and retrospective cohort studies, insurance claims and pharmacy benefit database analyses, national registry studies, systematic reviews and meta-analyses, and consensus documents from major scientific societies, provided they reported data relevant to one or more of the following: efficacy or effectiveness of GLP-1 RA therapy in adults; real-world adherence, persistence, or discontinuation; physiological, economic, or psychological barriers to long-term use; and nutritional, behavioral, or digital interventions intended to support persistence or mitigate adverse effects.

Exclusion criteria. Excluded were non–English-language publications, studies conducted exclusively in pediatric populations (<19 years), single case reports unless illustrating a novel mechanistic concept (e.g., the neuroimaging case study cited in Section 5.3 of the main manuscript), and non–peer-reviewed grey literature, except where explicitly labeled as company-reported real-world evidence and clearly identified as such in the text.

5. Supplementary searches

In addition to the structured PubMed search documented above, directed searches were performed in Embase and Google Scholar using the equivalent drug-class and outcome terms, primarily to identify pivotal real-world studies, large cohort analyses, and recent (2023–2026) primary publications. Reference lists of pivotal randomized trials, key narrative and systematic reviews, and consensus documents were hand-searched to identify additional relevant publications that may not have been captured by the database queries. Because this is a narrative review, comprehensive title-and-abstract screening of all identified records was not performed; instead, studies were selected for inclusion based on topical relevance to the predefined themes of the review, with priority given to landmark randomized controlled trials, large real-world cohort and claims-database analyses, and recent primary publications. The final reference list comprises 87 publications, as listed in the main manuscript.

6. Methodological limitations

The narrative design of this review carries inherent limitations that should be considered when interpreting the synthesis presented in the main manuscript. Study selection was based on topical relevance rather than on systematic screening of all identified records, and no formal risk-of-bias assessment, GRADE rating, or meta-analytic synthesis was performed. Persistence definitions, allowable gap thresholds (60 vs 90 days), follow-up durations, and data sources differ substantially across the cited real-world studies, which precludes direct quantitative cross-cohort comparison. The evidence base is also predominantly derived from high-income settings, limiting generalizability to low- and middle-income countries.
